# Efficacy of alumina nanoparticles as a controllable tool for mortality and biochemical parameters of *Culex pipiens*

**DOI:** 10.1038/s41598-023-46689-6

**Published:** 2023-11-10

**Authors:** Nehad M. El‑Barkey, Mostafa Y. Nassar, Aya H. El‑Khawaga, Aida S. Kamel, Mohamed M. Baz

**Affiliations:** 1 Entomology Department, Faculty of Science, Benha University, Benha, 13518 Egypt; 2 Chemistry Department, Faculty of Science, Benha University, Benha, 13518 Egypt; 3 Department of Chemistry, College of Science, King Faisal University, Al-Ahsa, Saudi Arabia

**Keywords:** Chemical biology, Ecology, Chemistry

## Abstract

Mosquitoes still pose a clear risk to human and animal health. Recently, nanomaterials have been considered one of the cost-effective solutions to this problem. Therefore, alumina nanoparticles (Al) were synthesized using an auto-combustion method, followed by calcination at 600 and 800 °C. Glucose (G) and sucrose (Su) were used as fuels and the combustion was performed at pH 2, 7, and 10. The as-synthesized Al_2_O_3_ nanoparticles were characterized by XRD, FTIR, SEM, and TEM. Alumina nanoparticles prepared using G and Su fuels at pH 7 and 800 °C (Al-G7-800 and Al-Su7-800) have crystallite sizes of 3.9 and 4.05 nm, respectively. While the samples (Al-G7-600 and Al-Su7-600) synthesized at pH 7 and 600 °C were amorphous. The prepared alumina nanoparticles were applied to the larval and pupal stages of *Culex pipiens*. The results showed that alumina nanoparticles cause higher mortality in the 1st larval instar than in all other larval instars and pupal stages of *Culex pipiens* after treatment at a high concentration of 200 ppm. Additionally, the larval duration after treatment with LC_50_ concentrations of alumina (Al-G7-800 and Al-Su7-800) was 31.7 and 23.6 days, respectively, compared to the control (13.3 days). The recorded data found that the content of glutathione-S-transferase, alkaline/acid phosphatase, β/α-esterase, and total protein were altered upon treatment with the LC_50_ concentration of alumina (Al-G7-800) nanoparticles. Based on these findings, alumina nanoparticles are a promising candidate as a potential weapon to control pests and mosquitoes.

## Introduction

Mosquitoes are of medical and veterinary importance; they are generally considered potential vectors for many protozoa, viruses, bacteria, and nematodes that threaten human and animal health with many diseases such as malaria, filariasis, yellow fever, dengue fever, and Japanese encephalitis in humans and bovine malaria and brucellosis in animals^1–4^. However, these diseases cause millions of deaths, long-term disabilities, and other effects that last a lifetime. In 2015 alone, there were an estimated 214 million new cases of malaria and 438,000 deaths. Malaria is a feverish illness that is caused by *Plasmodium spp.* parasites that are spread by *Anopheles spp*. Dengue has also spread 30 times faster around the world in the last 30 years^5^. In Central America in 2019, there were almost 20,000 cases of malaria due to local mosquitoes^6^.

Most of the people who get infected with these diseases live in developing countries in relatively inaccessible regions and have conditions that make them more likely to develop chronic infections. This includes rural and indigenous communities, poor women, and children^1,7,8^. Based on the high reproductive ability of mosquitoes and their ability to adapt to multiple environments, mosquitoes can transmit and spread diseases among the world's population, leading to the death of millions every year. The major problem of mosquitoes in disease transmission to either humans or animals is firmly rooted in their remarkable ability to spread and adapt to several aquatic breeding places and exploit large quantities of suspended food that are found in sources of stagnant water. Wherever water is stagnant, mosquitoes have a chance to reproduce, so any site where there is a collection of water whether permanent or temporary, is considered to be a suitable habitat for mosquito breeding. Among these places are pools, canals, ponds, ditches, irrigation channels, unused tires, and wells^9^.

The widespread house mosquito *Culex pipiens* is a major reason for the epidemics of Rift Valley fever that occurred in Egypt as well as the extensive transmission of Bancroft filariasis in the Nile Delta and is still a vital vector for many fevers in Egypt and the world^5,10^. Consequently, controlling vectors is at the forefront of innovative solutions that have been taken into account. However, synthetic insecticide applications, despite their high effectiveness against these species, these substances are problematic because of their frequent use and misuse, which in turn has led to environmental pollution, especially in agricultural and aquatic ecosystems, as well as developing resistance to almost all insecticide classes^11^. Therefore, insect resistance provokes an imperative need for developing new control tools and formulations.

Nanotechnology opens new horizons for scientific technology that could provide a cost-effective solution to a lot of challenging environmental problems. Remarkably, nanoparticles have been applied in many fields such as industry, biomedicine, and agriculture^12–14^, where nanoparticles have been recently used as pesticides against insects, either medically or agriculturally^15^. Metal oxide nanoparticles have received much attention and have been widely produced over the last few years in many fields^16,17^. Many researchers confirm the high efficacy of metal oxide nanoparticles as larvicides to mosquitoes and other insects^18,19^. Aluminum oxide nanoparticles are considered a very active member of the metal oxide nanomaterial family, as they can be readily handled and are easily accessible^20–22^. Considerably, alumina nanoparticles are characterized by exceptional properties, i.e., their simple and cost-effective protocols and easy surface functionalization. Therefore, they are widely used in the biological environment, particularly in biomedicine and biotechnology, including biosensing treatment of diseases, drug delivery, destruction of microbes^23^, insecticide formulation, and orthopedic applications^24^. This study sheds light on alumina nanoparticles as an alternative insecticide against mosquitoes and other insects.

The preparation of aluminum oxide nanoparticles will presumably be effective against mosquito larvae as an alternative to the use of synthetic insecticides to avoid environmental harm and insecticidal hazards. Therefore, the objective of this study is to fabricate aluminum oxide nanoparticles via a facile auto-combustion method and investigate the toxicity of the as-prepared alumina nanoparticles against *Culex pipiens* mosquito larvae and pupae. The alteration of some biological aspects will be estimated following treatment with sub-lethal concentrations of the tested aluminum oxide nanoparticles.

## Materials and methods

### Materials

Aluminum nitrate, glucose, sucrose, ammonia, TetraMin, and nitric acid (98%, density of 1.5 g/cm^3^) were supplied by El-Nasr Pharmaceutical Chemicals Company, Egypt. The bovine albumin standard was purchased from the Stanbio laboratory (Texas, USA). The Coomassie brilliant blue G-250 was from Sigma (Sigma Chemical Co.). P-nitro anisole (purity 97%) was obtained from Ubichem Ltd. (Hampshire), while nicotinamide adenine dinucleotide phosphate (the reduced form, NADPH), was obtained from BDH Chemicals Ltd. (Poole, England). The rest of the chemicals were of high quality and purchased from commercially established local companies. *Culex pipiens* mosquitoes were obtained from the Medical Entomology Section, Entomology Department, Faculty of Science, Benha University.

### *Culex pipiens* colony

*Culex pipiens* larvae were reared and kept as a continuous series of laboratory mosquito colonies at 27 ± 2 °C, 75 ± 5% RH, and a photoperiod of 14:10 h (light/dark) in the Medical Entomology Section, Entomology Department, Faculty of Science, Benha University. Larvae of *Culex pipiens* were fed on TetraMin food fish. TetraMin was added to ground bread with a ratio of 3:1 to prevent any fungal infection. As for pupae, they were periodically transferred from white enamel plates to white plastic cups containing de-chlorinated tap water that were put in screened wooden cages of dimensions (35 × 35 × 35 cm), where adults were allowed to emerge. Periodically, adults were provided with a 10% sucrose solution after a meal of blood through an anesthetized hamster mouse. Larvae and pupae were maintained under the same laboratory conditions^18^.

### Synthesis of aluminum oxide nanoparticles

Aluminum oxide nanoparticles were prepared by adding an aqueous solution of aluminum nitrate (10 mL, 10.25 mmol, 4.05 g) to an aqueous solution of glucose (G) as fuel (80 mL, 28 mmol, 9.2 g). The reaction solution was magnetically stirred for 5 min, then divided into four portions: the pH value of the first portion was left unadjusted, and the pH values of the other portions were adjusted into 2, 7, and 10, separately, using 0.2 M NaOH or HNO_3_ solutions. Afterward, each solution portion was subjected to auto-combustion at 300 °C on a hot plate for 10 min. Each produced burnt residue after auto-combustion was divided into two parts. One part was calcined at 600 °C for 3 h, and the other part at 800 °C for 3 h. Alumina nanoparticles were prepared at unadjusted and adjusted pH (2, 7, and 10, respectively) and calcined at 600 °C as well. These products are denoted by the symbols (Al-G-600, Al-G2-600, Al-G7-600, and Al-G10-600, respectively). Similar experiments were conducted using sucrose fuel (Su). In the case of sucrose fuel, the products calcined at 600 °C were denoted by the symbols (Al-Su-600, Al-Su2-600, Al-Su7-600, and Al-Su10-600, respectively). While the products calcined at 800 °C by glucose and sucrose fuels were denoted as Al-G-800, Al-G2-800, Al-G7-800, Al-G10-800, Al-Su-800, Al-Su2-800, Al-Su7-800, and Al-Su10-800, respectively.

### Characterization of aluminum oxide nanoparticles

Characterization is a crucial procedure to elucidate the crystallinity, phase purity, chemical structure, and morphology of the prepared aluminum oxide nanoparticles. Therefore, X-ray diffraction (XRD), Fourier transmission infrared spectroscopy (FT-IR), scanning electron microscopy (SEM), and transmission electron microscopy (TEM) were employed for this research to investigate the aforementioned characteristics, respectively.

### Larvicidal and pupicidal bioassay activity

The toxicity of the selected samples of alumina nanoparticles was assessed against the 1st, 2nd, 3rd, and 4th larval instars and pupal stage of *Culex pipiens* under laboratory conditions (27 ± 2 °C, 75 ± 5% RH). Stock dispersion was prepared by adding one gram of each selected sample to one liter of distilled water using an ultrasonicator to be equally dispersed for preparing different concentrations (5, 25, 50, 100, and 200 ppm). It is worth mentioning that twenty larvae per concentration were transferred to a glass beaker containing 250 mL of distilled water used for all experiments. The experiment was replicated three times with an untreated group. The mean mortality % was recorded after 24 and 48 h of the exposure period. The larval-adult duration of *Culex pipiens* was determined by LC_50_ concentration.

### Preparation of *Culex pipiens* larvae for biochemical analyses

The laboratory strain of the 4th larval instar of *Culex pipiens* was treated with the LC_50_ concentration of the tested alumina nanoparticles. Afterward, 0.5 to 1 g of treated larvae were frozen at − 25 °C for a week. Untreated larvae were maintained in the same conditions. Treated and untreated samples were transferred to the laboratory in iceboxes (− 20 °C) for biochemical analyses. Larval bodies were homogenized using buffer (1 g of insect body/1 mL) in a chilled glass-teflon tissue grinder (ST-2 Mechanic-Preczyina, Poland) for 3 min^25^. Afterward, homogenates were centrifuged at 14,000 rpm in a refrigerated centrifuge for 15 min at a temperature of − 2 °C. Then the supernatant was kept for 2 weeks at − 5 °C to conduct different biochemical analyses.

### Determination of biochemical parameters

#### Determination of nonspecific esterases for the 4th larval instar of *Culex pipiens*

Beta esterases (β-esterases) and alpha esterases (α-esterases) were evaluated using β-naphthyl acetate or α-naphthyl acetate as substrates, respectively. The reaction mixture was composed of 5 mL substrate solution (3 × 10–4 M β- or α-naphthyl acetate, 1% acetone, and 0.1 M phosphate buffer, pH 7) and 20 µL of larval homogenate. Then this mixture was subjected to incubation for 15 min at 27 °C, and then 1 mL of diazo blue color reagent (prepared by mixing 2 parts of 1% diazo blue B with 5 parts of 5% sodium lauryl sulfate). The developed color was read at 600 or 555 nm for β- and α-naphthol produced from the hydrolysis of the substrate, respectively. β- and α-naphthol standard curves were prepared by dissolving 20 mg β- or α-naphthol in 100 mL of phosphate buffer at pH 7 (stock solution). Afterward, ten milliliters of stock solution were diluted up to 100 mL by the buffer. Aliquots of 0.1, 0.2, 0.4, 0.8, and 1.6 mL of the diluted solution (equal to 2, 4, 8, 16, and 32 µg naphthol) were pipetted into test tubes and filled to 5 mL with phosphate buffer. One milliliter of diazo blue reagent was added, and the developed color was measured as mentioned before^26^.

#### Determination of glutathione S-transferase for the 4th larval instar of *Culex pipiens*

The reaction mixture was made up of 1 mL of the potassium salt of phosphate buffer (pH 6.5), 100 µL of Glutathione (GSH), and 200 µL of larval homogenate. The reaction started with the addition of 25 µL of the substrate 1‐chloro‐2, 4‐dinitrobenzene (CDNB) solution. The concentration of both GSH and CDNB was adjusted to be 5 mM and 1 mM, respectively. Enzyme and reagents were incubated at 30 °C for 5 min. The increment in absorbance at 340 nm was recorded against a blank containing everything except the enzyme to determine the nanomole substrate conjugated (min/larva) using a molar extinction coefficient of 9.6/mM/cm^27^.

#### Determination of phosphatases for the 4th larval instar of *Culex pipiens*

In this method, the phenol released by the enzymatic hydrolysis of disodium phenyl phosphate reacts with 4-amino antipyrine, and by the addition of potassium ferricyanide, the characteristic brown color is produced. The reaction mixture consisted of 1 mL carbonate buffer (pH 10.4) for alkaline phosphatase or 1 mL citric buffer (pH 4.9) for acid phosphatase, 1 mL of 0.01 M disodium phenyl phosphate (substrate), and 0.1 mL sample, which were mixed and incubated for exactly 30 min at 37 °C. At the end of the incubation period, 0.8 ml of 0.5 N NaOH was added to stop the reaction. Afterwards, 1.2 mL of 0.5 N NaHCO_3_ were added, followed by the addition of 1 mL of 4-amino antipyrine solution (1%) and 1 mL of potassium ferricyanide (0.5%). The produced color was measured immediately at 510 nm. The enzyme activity is expressed in units (U), where 1 unit will hydrolyze 1.0 µM of p-nitrophenyl phosphate per minute at 37 °C, and pH 10.4 and 4.8 for alkaline and acid phosphatases, respectively. All experiments involved 3–4 replicates (insects’ homogenates), and the results of biochemical determinations were pooled from triplicate determinations^28^.

#### Determination of total protein for the 4th larval instar of *Culex pipiens*

The protein reagent was prepared by dissolving 100 mg of Coomassie Brilliant Blue G-250 in 50 mL of 95% ethanol. Moreover, 100 mL of 85% (w/v) phosphoric acid was added to the previous solution. The resulting solution was diluted to a final volume of 1 L. Sample solution (50 µL) or for preparation of the standard curve, 50 µL of serial concentrations containing 10 to 100 µg bovine serum albumin were pipetted into test tubes. The volume in the test tube was adjusted to 1 mL with phosphate buffer (0.1 M, pH 6.6). Five millimeters of protein reagent were added to the test tube, and the contents were mixed either by inversion or vortexing. The absorbance at 595 nm was measured after 2 min against a blank prepared from 1 mL of phosphate buffer and 5 mL of protein reagent^29^.

### Statistical analysis

The statistical analysis was conducted using a one-way ANOVA test with five factors with a significance level of 0.05 for the whole set of results using SPSS (ver. 22). Data were treated as complete randomization designs^30^. Multiple comparisons were subjected to Fisher’s least significant difference (LSD). As for Lethal Concentration (LC_50_), it was calculated using Probit analysis software^31^. Mean percentages of larval mortality were calculated and corrected for natural mortalities; corrected mortality = (observed mortality % − control mortality %)/100 (control mortality × 100)^32^. The enzymes’ results were analyzed by one-way analysis of variance (ANOVA) using COSTA statistical software. When the ANOVA statistics were significant (P < 0.01), the means were compared by Duncan’s multiple range test.

### Institutional review board

The study was conducted according to the guidelines of the Declaration of Benha University and approved by the Ethics Committee of the Faculty of Science, Benha University under the Code: BUFS 2023-44Ent.

## Results and discussion

Nanotechnology is a new scientific technology that could provide a cost-effective solution to some of the most challenging environmental problems, as it is applied in numerous fields such as industry, biomedicine, and even agriculture^12^. Metal oxide nanoparticles have received much attention and have been widely produced in recent years^33^. Investigators have shed light on the general advantages of nanomaterials, recommending their safe application in pesticide production as an application of nanotechnology that will be useful to meet targeted delivery of pesticides. Unlike bulk materials, nanoparticles have unique physical and chemical properties such as tunable size that make them more effective in drug delivery systems^34^, lithium-ion batteries^35^ and insecticides^36^. At the nanoscale, size-dependent effects are more controlled. Therefore, alumina nanoparticles were employed to conduct this study.

**Figure 1:**
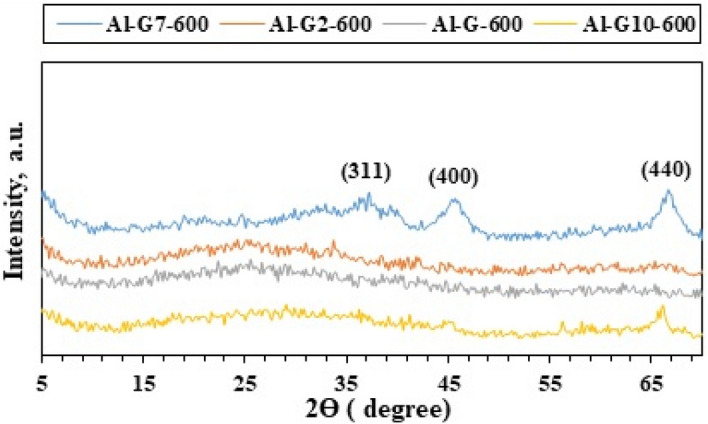
XRD patterns of Al_2_O_3_ nanoparticles; Al-G-600, Al-G2-600, Al-G7-600, Al-G10-600 prepared by auto-combustion method using glucose under conditions: unadjusted pH, pH 2, 7 and 10 respectively, and calcination at 600 °C for 3 h.

**Figure 2:**
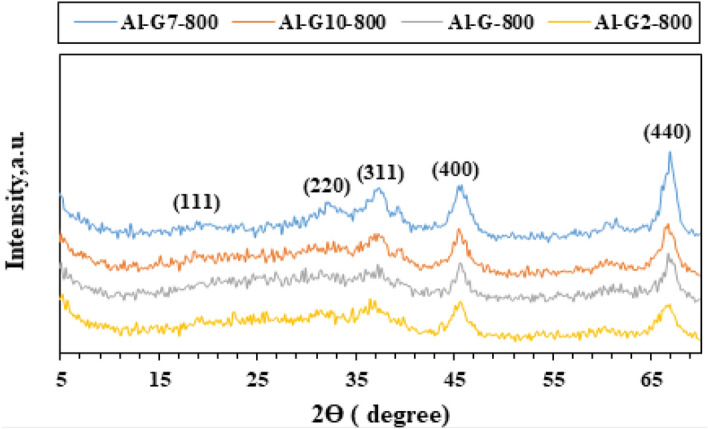
XRD patterns of Al_2_O_3_ nanoparticles; Al-G-800, Al-G2-800, Al-G7-800, Al-G10-800 prepared by auto-combustion method using glucose under conditions: unadjusted pH, pH 2, 7 and 10 respectively, and calcination at 800 °C for 3 h.

**Figure 3:**
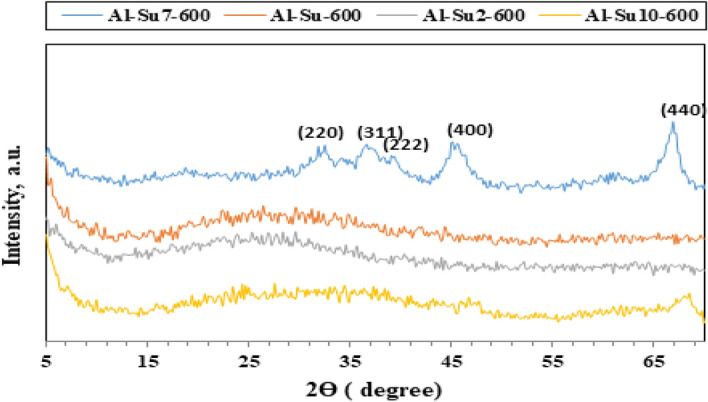
XRD patterns of Al_2_O_3_ nanoparticles; Al-Su-600, Al-Su2-600, Al-Su7-600, Al-Su10-600 prepared by auto-combustion method using sucrose fuel under conditions: unadjusted pH, pH 2, 7 and 10 respectively, and calcination at 600 °C for 3 h.

**Figure 4:**
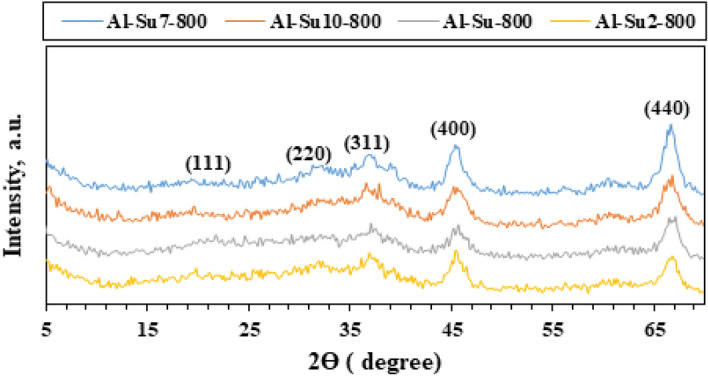
XRD patterns of Al_2_O_3_ nanoparticles; Al-Su-800, Al- Su2-800, Al- Su7-800, Al- Su10-800 prepared by auto-combustion method using sucrose fuel under conditions: unadjusted pH, pH 2, 7 and 10 respectively, and calcination at 800 °C for 3 h.

The X-ray diffraction analysis (XRD) technique was used to investigate the phase composition, purity, and crystallinity of the as-synthesized Al_2_O_3_ nanoparticles. Other phases were not detected, which emphasized that the Al_2_O_3_ nanoparticles phase was pure with no impurities. XRD patterns (Figs. 1, 2, 3, 4) show that Al_2_O_3_ nanoparticles prepared by adding glucose and sucrose fuels at pH 7 and calcined at 600 and 800 °C are of cubic phase and well-indexed [No. 01-010-0425; space group: Fd3m]^37^. However, alumina nanoparticles (Al-G7-800 and Al-Su7-800) were more crystalline and had crystallite sizes of 3.90 and 4.05 nm, respectively. While the other products, Al-G7-600 and Al-Su7-600, are amorphous. This may be due to the important role of temperature in crystallization, as higher calcination temperatures facilitate the growth of crystals^38,39^. The crystallite size was calculated using the Debye–Scherrer equation^40^:

$${\text{D}} = 0.9\lambda /\beta \;\cos \theta_{{\text{B}}}$$

where λ (nm) is the X-ray radiation wavelength, β is the full width of the diffraction peak at half maximum (FWHM), and θ_B_ is the Bragg diffraction angle. The results showed that calcining alumina at 600 and 800 °C was sufficient for obtaining pure nano alumina products, while calcining the samples at lower temperatures produced less crystalline or amorphous alumina contaminated with carbon residues^41^. Conversely, it was reported that well-crystallized α-Al_2_O_3_ nanoparticles were prepared after calcination at 1100 °C^42^. Therefore, this study implied that pure and crystalline alumina nanoparticles were obtained at a lower temperature.

Glucose and sucrose fuels were employed in the production of alumina nanoparticles. The results indicated that the usage of glucose and sucrose fuels at pH 7 produced smaller crystallites, finer agglomeration, and a higher specific surface area than those synthesized by utilizing different pH values. Using glucose as a fuel was better than sucrose. This may be because the nature of agglomeration is primarily governed by the enthalpy or flame temperature generated and the amount of gases that escape during combustion, and subsequently, they affect different characteristics such as particle size, surface area, and crystallinity^42,43^. So, fuel type and pH value are important parameters to the combustion process^44^. It was detected that the fuel affected the properties of alumina nanopowders, which were prepared by an auto-combustion synthesis using aluminum nitrate as an oxidizer and urea ammonium acetate and ammonium nitrate as fuels^38^.

Fuel’s impact on the properties of alumina nanoparticles may be attributed to the reduction of the exothermicity of the combustion reaction and the nature of the reaction between both the oxidizing agent and the fuel. This finding was in agreement with data published earlier which used two different fuels, ovalbumin and urea, along with aluminum format to prepare alpha-alumina nanoparticles of different sizes ranging 20–40 nm.

**Figure 5:**
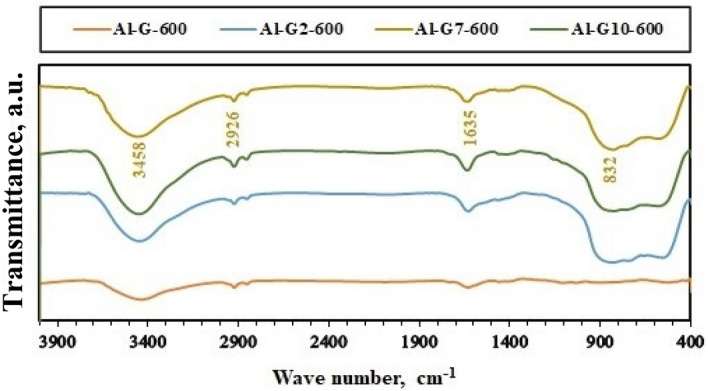
FT-IR spectra of Al_2_O_3_ nanoparticles; Al-G-600, Al-G2-600, Al-G7-600, Al-G10-600 prepared by auto-combustion method using glucose under conditions: unadjusted pH, pH 2, 7 and 10 respectively, and calcination at 600 °C for 3 h.

**Figure 6:**
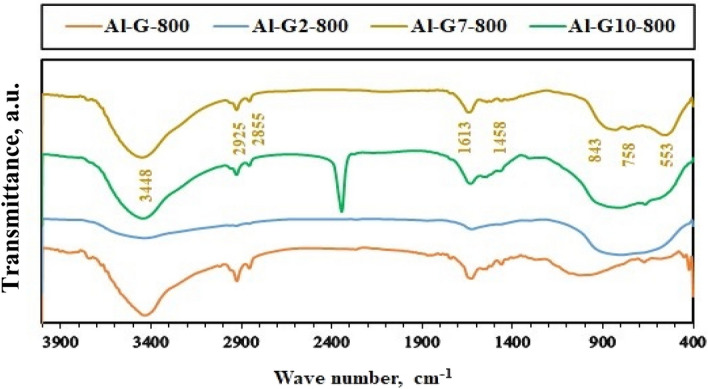
FT-IR spectra of Al_2_O_3_ nanoparticles; Al-G-800, Al-G2-800, Al-G7-800, Al-G10-800 prepared by auto-combustion method using glucose under conditions: unadjusted pH, pH 2, 7 and 10 respectively, and calcination at 800 °C for 3 h.

**Figure 7:**
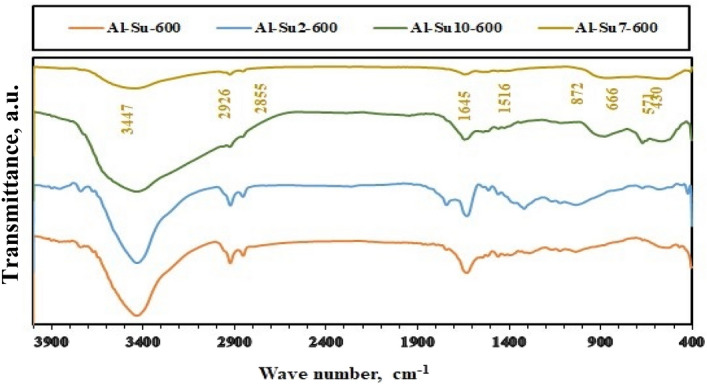
FT-IR spectra of Al_2_O_3_ nanoparticles; Al-Su-600, Al-Su2-600, Al-Su7-600, Al-Su10-600 prepared by auto-combustion method using sucrose under conditions: unadjusted pH, pH 2, 7 and 10 respectively, and calcination at 600 °C for 3 h.

**Figure 8:**
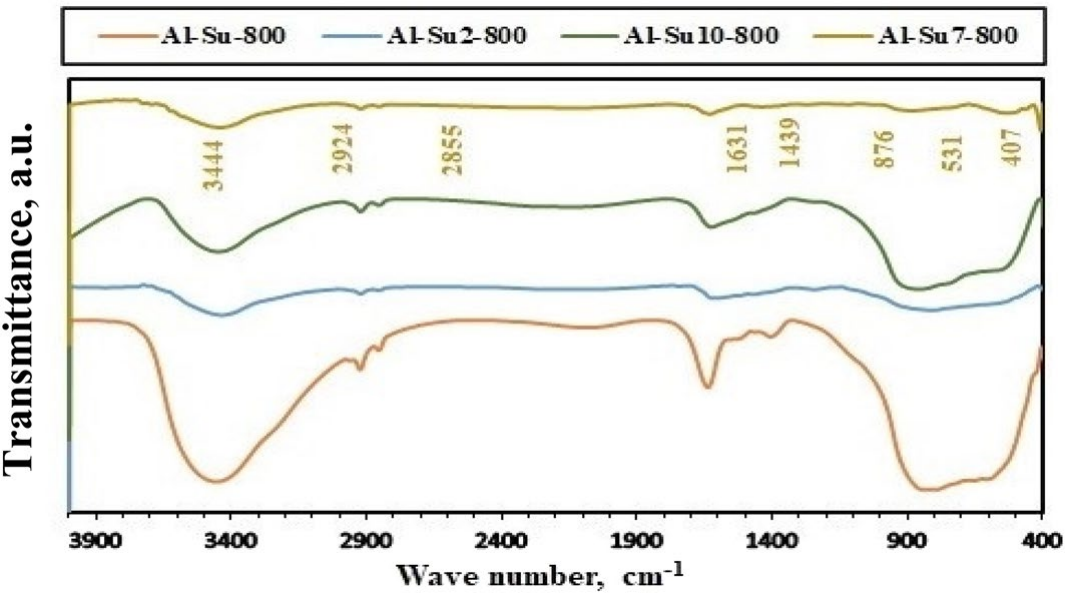
FT-IR spectra of Al_2_O_3_ nanoparticles; Al-Su-800, Al-Su2-800, Al-Su7-800, Al-Su10-800 prepared by auto-combustion method using sucrose under conditions: unadjusted pH, pH 2, 7 and 10 respectively, and calcination at 800 °C for 3 h.

On the other hand, the chemical composition of the calcined samples was identified using FT-IR spectroscopy, and the results are shown in Figs. 5, 6, 7 and 8. The FT-IR spectra indicated the composition of alumina nanoparticles (Al-G7-600, Al-Su7-600, Al-G7-800, and Al-Su7-800) by exhibiting the corresponding characteristic frequencies in the region of 400–850 cm^−1^. On the contrary, the other samples prepared using other pH values revealed additional vibrational bands due to the carbon residues. The band that appeared at ca. 800–1000 cm^−1^ may be due to the Al-O-C bond between Al^3+^ and glucose and sucrose fuels. The band that appeared at ca. 1339–1645 cm^−1^ indicated alumina formation. While the band at ca. 2855–2945 cm^-1^ is probably due to stretching C-H bond of the remaining carbon residue. Finally, the band that appeared at ca. 3444–3454 cm^−1^ may be due to (–OH) stretching vibrations that indicate the presence of a hydroxyl group^38,45^.

**Figure 9:**
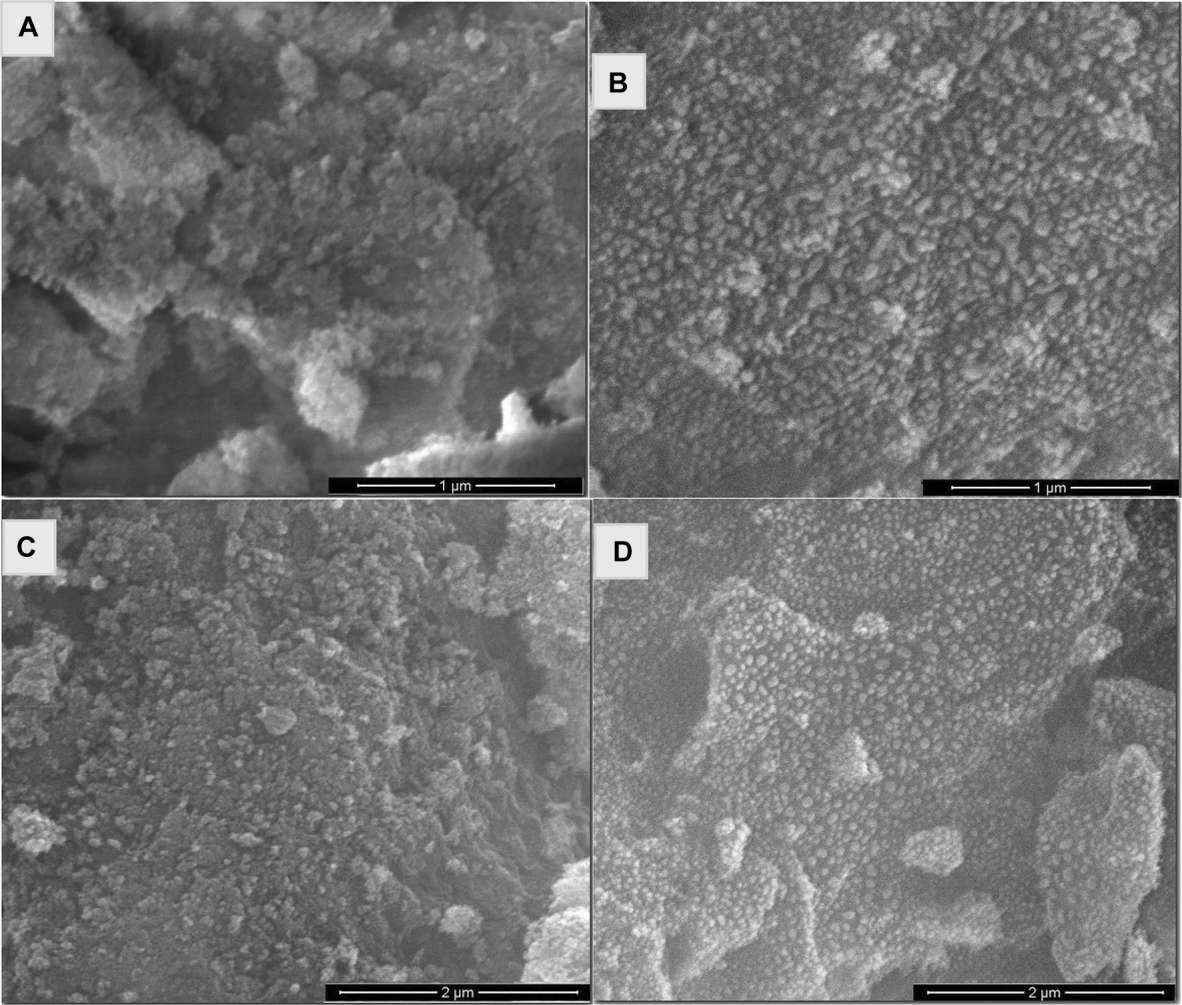
SEM images of Al_2_O_3_ nanoparticles prepared by auto-combustion method using glucose fuel ((Al-G7-600) (**a**) and (Al-G7-800) (**b**)) and sucrose fuel ((Al-Su7-600) (**c**) and (Al-Su7-800) (**d**)) at pH 7 and calcined at 600 and 800 °C for 3 h.

**Figure 10:**
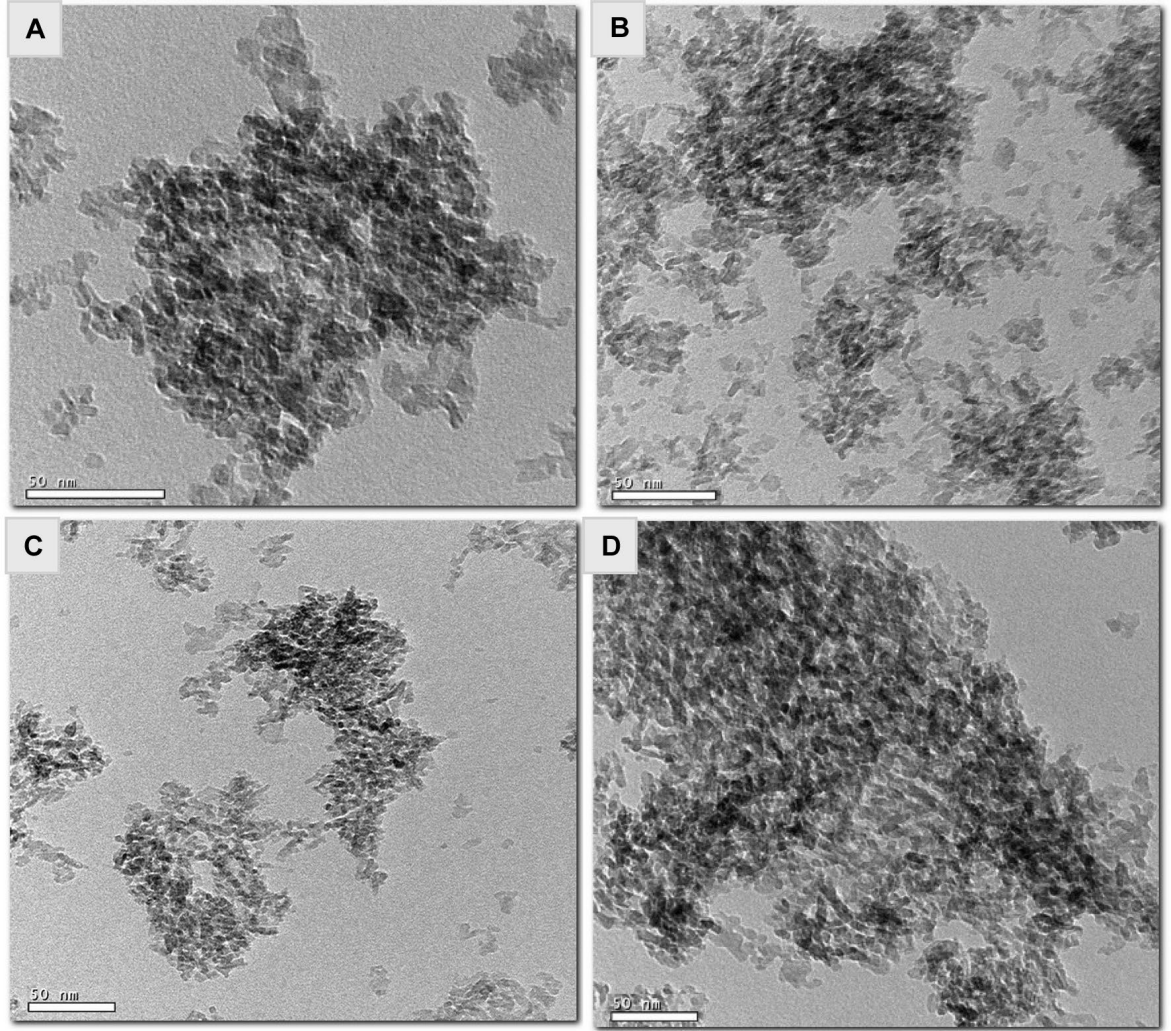
TEM images of Al_2_O_3_ nanoparticles prepared by auto-combustion method using glucose fuel ((Al-G7-600) (**a**) and (Al-G7-800) (**b**)) and sucrose fuel ((Al-Su7-600) (**c**) and (Al-Su7-800) (**d**)) at pH 7 and calcined at 600 and 800 °C for 3 h.

The surface morphology of the as-prepared Al_2_O_3_ (Al-G7-600, Al-G7-800, and Al-Su7-600 and Al-Su7-800) was investigated by scanning electron microscope (SEM), and the images are exhibited in Fig. 9. The investigation of the SEM images of the products revealed that they are composed of agglomerates of spherical and irregular morphological shapes. Besides, they are also porous. The products calcined at 800 °C have more spherical shapes than the products calcined at 600 °C. Additionally, the products obtained using sucrose fuel have more symmetrical spherical particles than those obtained using glucose fuel. In addition, the microstructure of the prepared nanomaterials was further characterized by transmission electron microscopy (TEM), as shown in Fig. 10. The TEM images of the products exhibited that the particles have spherical, cubic, and irregular shapes with an average particle size of 15 nm which is consistent with the XRD results. Moreover, the TEM images revealed that alumina (Al-G7-800 and Al-G7-600) synthesized using glucose fuel was more porous than alumina (Al-Su7-800 and Al-Su7-600) synthesized using sucrose fuel. This is probably because the combustion reaction is more exothermic and gives off more CO_2_, N_2_, and H_2_O gases, resulting in more porous products^41^.

The mortality of *Culex pipiens* larval and pupal stages treated with alumina nanoparticles was investigated. The results showed that alumina nanoparticles induced mortality in all *Culex pipiens* larval and pupal stages (Tables 1, 2, 3, 4). These results might be explained as nanoparticles being small enough to penetrate nearly all the body whatever the path they took, either by inhalation or feeding. Moreover, insect mortality could be interpreted in terms of protein expression and its relation to RNA and DNA synthesis. It is worth noting that nanoparticles may attenuate the expression of proteins, causing inhibition of RNA and DNA synthesis, which were considered the first signs of cell death^46^. In addition, alumina nanoparticles (Al-G7-800, Al-Su7-800) calcined at 800 °C using glucose and sucrose fuels at pH 7 were more effective on *Culex pipiens* larval and pupal stages than alumina (Al-G7-600, Al-Su7-600) calcined at 600 °C at the same conditions. This may be because alumina (Al-G7-800, Al-Su7-800) nanoparticles have a smaller particle size and higher surface area than alumina particles (Al-G7-600, Al-Su7-600) coated with carbon residue layers^47^. Distinctly, the present study exhibited that the highest mortality was observed in the 1st larval instar when compared to other larval instars and pupal stages treated with alumina (Al-Su7-600), where the mortalities were 85% and 95%, and with alumina (Al-G7-600), the mortalities were 93.33% and 100% at 200 ppm concentration after 24 h and 48 h, respectively. Meanwhile, in the 1st larval instars treated with Al-Su7-800, the mortality was 95% and 100% after 24 h and 48 h, respectively, and in the 1st larval instars treated with Al-G7-800, they were 100% after both 24 h and 48 h.

**Table 1:** Toxicity of alumina nanoparticles (Al-Su7-600) against larvae and pupae of *Culex pipiens,* 24 and 48 h post-treatment.

Time (h)	Conc. (ppm)	Mean mortality % of *Culex pipiens* larval and pupal stages (± SD)					Total mean (%)
		1st	2nd	3rd	4th	Pupal stage	
24	Control	0 ± 0fA	0 ± 0fA	0 ± 0fA	0 ± 0eA	0 ± 0eA	0 ± 0f
	5	10.00 ± 2.89eA	6.67 ± 1.67eB	3.33 ± 1.67eC	1.67 ± 1.67eCD	0 ± 0eD	4.33 ± 1.18e
	25	23.33 ± 4.41dA	18.33 ± 1.67 dB	11.67 ± 1.67dC	6.67 ± 3.33dD	3.33 ± 1.67dE	12.67 ± 2.23d
	50	36.67 ± 4.41cA	30.00 ± 5.00cB	21.67 ± 4.41cC	10.00 ± 2.89cD	6.67 ± 1.67cE	21.00 ± 3.39c
	100	60.00 ± 5.00bA	46.67 ± 6.01bB	35.00 ± 5.00bC	23.33 ± 3.33bD	18.33 ± 1.67bE	36.67 ± 4.41b
	200	85.00 ± 2.89aA	71.67 ± 6.01aB	61.67 ± 6.01aC	40.00 ± 5.77aD	30.00 ± 8.66aE	57.67 ± 5.87a
48	Control	0 ± 0fA	0 ± 0fA	0 ± 0fA	0 ± 0fA	1.67 ± 1.67eA	0.33 ± 0.33f
	5	18.33 ± 1.67eA	13.33 ± 1.67eB	8.33 ± 3.33eC	5.00 ± 2.89eC	1.67 ± 1.67eD	9.33 ± 1.82e
	25	31.67 ± 1.67dA	26.67 ± 4.41 dB	20.00 ± 5.00dC	15.00 ± 2.89dD	5.00 ± 2.89dE	19.67 ± 2.82d
	50	50.00 ± 11.55cA	45.00 ± 5.77cB	31.67 ± 1.67cC	26.67 ± 4.41cD	18.33 ± 3.33cE	34.33 ± 3.93c
	100	71.67 ± 9.28bA	60.00 ± 7.64bB	45.00 ± 5.77bC	38.33 ± 8.82bD	31.67 ± 3.33bE	49.33 ± 4.78b
	200	95.00 ± 5.00aA	85.00 ± 2.89aB	70.00 ± 5.77aC	63.33 ± 6.01aD	48.33 ± 10.14aE	72.33 ± 5.00a

Where, a, b, and c symbols mean that there is no significant difference (P > 0.05) between any two means, within the same column that has the same superscript letter. Besides, A, B, and C symbols mean that there is no significant difference (P > 0.05) between any two means for the same attribute, within the same row have the same superscript letter.

**Table 2:** Toxicity of alumina nanoparticles (Al-G7-600) against larvae and pupae of *Culex pipiens* 24 and 48 h post-treatment.

Time (h)	Conc. (ppm)	Mean mortality % of *Culex pipiens* larval and pupal stages (± SD)					Total mean (%)
		1st	2nd	3rd	4th	Pupae	
24	Control	0 ± 0fA	0 ± 0fA	0 ± 0fA	0 ± 0fA	0 ± 0eA	0 ± 0f
	5	13.33 ± 1.67eA	11.67 ± 1.67eA	6.67 ± 1.67eB	3.33 ± 1.67eC	0 ± 0eD	7.00 ± 1.45e
	25	31.67 ± 3.33dA	21.67 ± 4.41 dB	18.33 ± 1.67dC	13.33 ± 1.67dD	3.33 ± 1.67dE	17.67 ± 2.71d
	50	56.67 ± 6.01cA	46.67 ± 4.41cB	31.67 ± 4.41cC	21.67 ± 1.67cD	8.33 ± 4.41cE	33.00 ± 4.90c
	100	75.00 ± 5.77bA	60.00 ± 5.77bB	43.33 ± 6.01bC	31.67 ± 4.41bD	23.33 ± 1.67bE	46.67 ± 5.36b
	200	93.33 ± 4.41aA	85.00 ± 2.89aB	75.00 ± 2.89aC	65.00 ± 5.77aD	38.33 ± 3.33aE	71.33 ± 5.31a
48	Control	0 ± 0fA	0 ± 0fA	0 ± 0fA	0 ± 0fA	0 ± 0fA	0 ± 0f
	5	23.33 ± 4.41eA	18.33 ± 3.33eB	16.67 ± 1.67eC	10.00 ± 2.89eD	3.33 ± 1.67eE	14.33 ± 2.17e
	25	46.67 ± 4.41dA	31.67 ± 4.41 dB	30.00 ± 5.77dC	21.67 ± 4.41dD	8.33 ± 1.67dE	27.67 ± 3.74d
	50	65.00 ± 10.00cA	58.33 ± 8.82cB	43.33 ± 4.41cC	36.67 ± 3.33cD	23.33 ± 4.41cE	45.33 ± 4.74c
	100	88.33 ± 6.01bA	76.67 ± 6.01bB	60.00 ± 5.00bC	51.67 ± 3.33bD	35.00 ± 2.89bE	62.33 ± 5.32b
	200	100.00 ± 0.00aA	95.00 ± 2.89aB	86.67 ± 6.01aC	75.00 ± 2.89aD	50.00 ± 2.89aE	81.33 ± 4.94a

Where, a, b, and c symbols mean that there is no significant difference (P > 0.05) between any two means, within the same column that has the same superscript letter. Besides, A, B, and C symbols mean that there is no significant difference (P > 0.05) between any two means for the same attribute, within the same row have the same superscript letter.

**Table 3:** Toxicity of alumina nanoparticles (Al-Su7-800) against larvae and pupae of *Culex pipiens* 24 and 48 h post-treatment.

Time (h)	Conc. (ppm)	Mean mortality % of *Culex pipiens* larval and pupal stages (± SD)					Total mean (%)
		1st	2nd	3rd	4th	Pupae	
24	Control	0 ± 0fA	0 ± 0fA	0 ± 0fA	0 ± 0fA	0 ± 0eA	0 ± 0f
	5	18.33 ± 1.67eA	16.67 ± 4.41eB	10.00 ± 2.89eC	6.67 ± 1.67eD	1.67 ± 1.67eE	10.67 ± 1.94e
	25	40.00 ± 2.89dA	30.00 ± 2.89 dB	23.33 ± 3.33dC	15.00 ± 2.89dD	6.67 ± 1.67dE	23.00 ± 3.27d
	50	68.33 ± 3.33cA	53.33 ± 7.26cB	38.33 ± 1.67cC	25.00 ± 2.89cD	15.00 ± 2.89cE	40.00 ± 5.35c
	100	80.00 ± 2.89bA	70.00 ± 5.77bB	60.00 ± 2.89bC	46.67 ± 3.33bD	28.33 ± 3.33bE	57.00 ± 5.04b
	200	95.00 ± 2.89aA	95.00 ± 2.89aA	85.00 ± 2.89aB	65.00 ± 5.77aC	41.67 ± 3.33aD	76.33 ± 5.66a
48	Control	0 ± 0fA	0 ± 0fA	0 ± 0fA	0 ± 0fA	0 ± 0fA	0 ± 0f
	5	28.33 ± 1.67eA	26.67 ± 1.67eB	20.00 ± 2.89eC	15.00 ± 2.89eD	5.00 ± 2.89eE	19.00 ± 2.45e
	25	51.67 ± 6.01dA	50.00 ± 5.00dA	36.67 ± 4.41 dB	25.00 ± 2.89dC	13.33 ± 1.67dD	35.33 ± 4.24d
	50	71.67 ± 8.82cA	71.67 ± 6.01cA	53.33 ± 4.41cB	43.33 ± 8.82cC	28.33 ± 1.67cD	53.67 ± 5.11c
	100	91.67 ± 4.41bA	86.67 ± 7.26bB	70.00 ± 2.89bC	60.00 ± 7.64bD	40.00 ± 2.89bE	69.67 ± 5.40b
	200	100.00 ± 0.00aA	98.33 ± 1.67aA	90.00 ± 5.77aB	83.33 ± 6.01aC	60.00 ± 2.89aD	86.33 ± 4.15a

Where, a, b, and c symbols mean that there is no significant difference (P > 0.05) between any two means, within the same column having the same superscript letter. Besides, A, B, and C symbols mean that there is no significant difference (P > 0.05) between any two means for the same attribute, within the same row having the same superscript letter.

**Table 4:** Toxicity of alumina nanoparticles (Al-G7-800) against larvae and pupae of *Culex pipiens* 24 and 48 h post-treatment.

Time (h)	Conc. (ppm)	Mean mortality % of *Culex pipiens* larval and pupal stages (± SD)					Total mean (%)
		1st	2nd	3rd	4th	Pupae	
24	Control	0 ± 0eA	0 ± 0fA	0 ± 0fA	0 ± 0fA	0 ± 0fA	0 ± 0f
	5	38.33 ± 4.41dA	23.33 ± 3.33eB	15.00 ± 2.89eC	10.00 ± 2.89eD	3.33 ± 1.67eE	18.00 ± 3.44e
	25	66.67 ± 4.41cA	56.67 ± 7.26 dB	43.33 ± 6.01dC	28.33 ± 1.67dD	10.00 ± 2.89dE	41.00 ± 5.69d
	50	90.00 ± 2.89bA	75.00 ± 2.89cB	61.67 ± 4.41cC	45.00 ± 2.89cD	20.00 ± 2.89cE	58.33 ± 6.59c
	100	100.00 ± 0.00aA	96.67 ± 1.67bB	85.00 ± 2.89bC	83.33 ± 4.41bD	35.00 ± 2.89bE	80.00 ± 6.34b
	200	100.00 ± 0.00aA	100.00 ± 0.00aA	100.00 ± 0.00aA	98.33 ± 1.67aA	50.00 ± 2.89aB	89.67 ± 5.33a
48	Control	0 ± 0dA	0 ± 0eA	0 ± 0eA	0 ± 0eA	0 ± 0fA	0 ± 0f
	5	53.33 ± 6.01cA	35.00 ± 2.89 dB	26.67 ± 1.67dC	21.67 ± 3.33dD	10.00 ± 2.89eE	29.33 ± 4.11e
	25	88.33 ± 4.41bA	78.33 ± 4.41cB	60.00 ± 5.77cC	50.00 ± 2.89cD	18.33 ± 1.67dE	59.00 ± 6.69d
	50	100.00 ± 0.00aA	95.00 ± 2.89bB	88.33 ± 4.41bC	81.67 ± 6.01bD	40.00 ± 2.89cE	81.00 ± 5.90c
	100	100.00 ± 0.00aA	100.00 ± 0.00aA	100.00 ± 0.00aA	98.33 ± 1.67aA	51.67 ± 4.41bB	90.00 ± 5.19b
	200	100.00 ± 0.00aA	100.00 ± 0.00aA	100.00 ± 0.00aA	100.00 ± 0.00aA	70.00 ± 2.89aB	94.00 ± 3.24a

Where, a, b, and c symbols mean that there is no significant difference (P > 0.05) between any two means, within the same column having the same superscript letter. Besides, A, B, and C symbols mean that there is no significant difference (P > 0.05) between any two means for the same attribute, within the same row having the same superscript letter.

Similarly, in another study, it was reported that the mortality of the *Aedes aegypti* mosquito was positively correlated with the concentration of silver nanoparticles^48^. Furthermore, larvicidal activity against *Anopheles stephensi* (80 ± 13.69%), *Culex quinquefasciatus* (72 ± 13.04%), and *Aedes aegypti* (65 ± 8.66%) mosquito larvae was reported after the previously mentioned species were treated with copper oxide nanoparticles^49^. This high mortality in the first larval instar may be due to their susceptibility caused by the declining rate of detoxification enzymes^50^.

Biochemical studies were also conducted and were presented in Table 5. This investigation was performed through the assessment of some biochemical parameters such as total protein, α-/β-esterases, glutathione transferases (GSTs), and alkaline and acid phosphatases after treating with the LC_50_ of alumina nanoparticles (Al-G7-800 and Al-Su7-800) that induce more mortality to investigate the reasons for mortality as indicated in Table 5. In the current results, total protein content is remarkably higher in two alumina nanostructures. This obtained result agreed with a study conducted earlier that indicated histological sections displaying that negatively charged gold nanoparticles were encapsulated inside the discoid cockroach nervous system, which indicated the depletion of proteins for forming a protein corona around the negatively charged gold nanoparticles as a type of defense mechanism^51^. This may be attributed to the active interaction between gold nanoparticles and proteins found within the biological system, including the nervous system^19^. These findings might point out that developing resistance to metal oxide nanoparticles is possible where the nanoparticles (below 50 nm in diameter) are small enough to be encapsulated^52^.

**Table 5:** Determination of total protein, alkaline phosphatase, acid phosphatase, β-esterases, GST, and α-esterases of 4th larval instar of *Culex pipiens* treated with LC_50_ of alumina nanoparticles (Al-G7-800 and Al-Su7-800) after 24 h post-treatment.

Sample	Enzymes activities (U × 103/g.b.wt) ± SD					
	Total protein	Alkaline phosphatase	Acid phosphatase	α-esterase	β-esterase	GST
Control	46.00 ± 0.55c	488.00 ± 8.33a	136.67 ± 1.20c	3740.33 ± 30.44a	772.00 ± 3.06c	449.67 ± 8.82a
Al-Su7-800	48.50 ± 0.98b	173.33 ± 1.67c	146.33 ± 0.88b	1712.33 ± 41.14b	848.00 ± 5.03b	281.00 ± 3.51c
Al-G7-800	52.67 ± 0.48a	224.67 ± 11.70b	176.33 ± 2.96a	1498.00 ± 45.92c	882.33 ± 4.84a	324.00 ± 8.02b

Where, a, b, and c symbols mean that there is no significant difference (P > 0.05) between any two means, within the same column having the same superscript letter. Besides, A, B, and C symbols mean that there is no significant difference (P > 0.05) between any two means for the same attribute, within the same row having the same superscript letter.

The tabulated data showed that α-esterase and glutathione transferase (GST) enzymes increased significantly in treated samples compared to untreated samples, indicating that these enzymes increased in *Culex pipiens* larvae as a defense mechanism against the effectiveness of Al_2_O_3_ nanoparticles^53^. Similar results reported that α-/β-esterases and glutathione transferase (GST) have been comprehensively scouted in different mosquito species^54^. The aforementioned enzymes may have developed resistance through direct detoxification and urge endogenous and exogenous compounds to be eliminated through different metabolic pathways and/or transformed into useless substances that an insect's body could easily get rid of^55^. Meanwhile, β-esterase activity took a reverse route on treatment with the LC_50_ of alumina nanoparticles, as it remarkably decreased. It is noteworthy that β-esterase activity often contradicts the activity of α-esterase^56^. This may be attributed to esterase proteins that are not completely similar in their primary DNA sequences and consequently differ in substrate specificities. Therefore, the catalytic site and binding site that comprise the enzyme’s active site are different in both α-/β-esterases^57,58^. Acid phosphatase is a lysosomal enzyme that is highly abundant in Malpighian tubules, guts, and disintegrating organs and tissues that have undergone cytolysis^59^. This enzyme is responsible for the hydrolysis of orthophosphate esters and transphosphorylation reactions to provide a phosphate pool for the synthesis of compounds of higher energy such as adenosine triphosphate (ATP), ATPase, and genetic materials (DNA or RNA)^60^. Significantly, in this study, alkaline and acid phosphatases were reduced in samples treated with the LC_50_ of alumina nanoparticles compared to the control samples. This highly significant decline may be due to a reduction in the lysosomal enzyme acid phosphatase caused by the ingestion of xenobiotics or toxic substances, which can passively affect the lysosome's activity or cause a lack of energy required for different vital functions and genetic malformations^61^.

Alkaline phosphatase is found primarily in intestinal epithelial tissues, and its core function is to supply phosphate ions from mononucleotides and ribonucleoproteins for different kinds of metabolic processes. However, alkaline phosphatase is active primarily in tissues with active membrane transport, such as intestinal epithelial cells, Malpighian tubules, and hemolymph^62^. Therefore, the remarkable reduction of alkaline phosphatase may be due to the binding of Al_2_O_3_ nanoparticles to the active sites of the enzyme. In addition, alkaline phosphatase may act as a hydrolase during the final stages of digestion, gonad maturation, and metamorphic molts^63^. Consequently, the prolonged larval-adult duration after treatment with the LC_50_ of alumina nanoparticles (Al-G7-800 and Al-S7-800) compared with the control shown in Table 6 may be owing to the remarkable reduction in alkaline phosphatases, which play a prominent role in molting, metabolism, cell signaling, and other physiological processes^61^.

**Table 6:** Means of larval and pupal duration (days) of *Culex pipiens* at LC_50_ of alumina (Al-G7-800 & Al-Su7-800) nanoparticles after 24 h.

Treatment	Larval and pupal stage											
	1st		2nd		3rd		4th		Pupae		Larval -Adult	
	Duration	Survival	Duration	Survival	Duration	Survival	Duration	Survival	Duration	Survival	Duration	Survival
Control	2.0 ± 0.0cC	96.0 ± 2.3aA	2.5 ± 0.3cB	94.6 ± 1.3aA	2.90 ± 0.15cA	97.3 ± 1.3aA	3.2 ± 0.2cA	94.6 ± 1.3aA	3.2 ± 0.21cA	98.6 ± 1.3aA	13.3 ± 0.7c	96.3 ± 07a
Al-G7-800	3.9 ± 0.4aD	54.6 ± 2.6bD	5.6 ± 0.5aC	62.6 ± 3.5bC	7.4 ± 0.55aB	76.0 ± 4.6bB	9.2 ± 0.3aA	81.3 ± 1.3bA	9.2 ± 0.31aA	86.6 ± 3.5bA	31.7 ± 2.5a	72.2 ± 3.4b
Al-Su7-800	3.1 ± 0.0bD	41.3 ± 3.5cD	3.9 ± 0.5bC	45.3 ± 4.8cD	4.83 ± 0.38bB	56.0 ± 2.31cC	7.4 ± 0.7bA	65.3 ± 1.3cB	7.4 ± 0.7bA	73.3 ± 5.8cA	23.6 ± 2.1b	56.3 ± 3.5c

Where, a, b, and c symbols mean that there is no significant difference (P > 0.05) between any two means, within the same column that has the same superscript letter. Besides, A, B, and C symbols mean that there is no significant difference (P > 0.05) between any two means for the same attribute, within the same row have the same superscript letter.

## Conclusions

In this study, mosquito larvae (*Culex pipiens*) were used to test how well alumina nanoparticles kill insects and how they affect biochemical parameters in the lab. This study sheds light on the mechanism of action of alumina nanoparticles and their impact on mosquito larvae. Alumina nanoparticles caused some developmental and enzymatic alterations in *Culex pipiens* larvae, demonstrating their insecticidal activity. Also, this kind of formulation could help cut down on the use of chemical pesticides, which are a major source of pollution and are making insects of medical importance such as *Culex pipiens* and *Anopheles spp.* resistant to them. In anticipation of the need for effective integration into pest management strategies, nano-based insecticides may be used in the fight against different insects that cause medical and economic problems without disturbing the environmental balance. Based on these results, alumina nanoparticles should be studied further, including an assessment of any effects on non-target biota.

## Data Availability

The datasets used and/or analyzed during the current study are available from the corresponding author on a reasonable request.
